# Editorial

**DOI:** 10.1007/s40955-022-00231-9

**Published:** 2022-11-01

**Authors:** Marion Fleige, Aiga von Hippel

**Affiliations:** 1 Deutsches Institut für Erwachsenenbildung – Leibniz-Zentrum für Lebenslanges Lernen, Bonn, Deutschland; 2 Humboldt-Universität zu Berlin, Berlin, Deutschland

## Inhaltliche Hinführung

Die Programmplanung hat eine grundlegende Bedeutung für die Erwachsenen- und Weiterbildung und das Zustandekommen von Unterrichtssituationen und bedarf im Sinne der Professionalisierung einer wissenschaftlich gestützten Dokumentation. Dabei stellen ‚Programme‘ den größeren konzeptionellen Rahmen von Angeboten dar und benötigen weitergehende pädagogische- und Gestaltungskompetenzen bzw. eine entsprechenden Reflexion. Umgekehrt sind über Programme Auslegungen von Bildung seitens der Pädagoginnen und Pädagogen, aber auch in der Gesellschaft, bei Trägerinstitutionen sowie auch bei Adressatinnengruppen rekonstruierbar (vgl. von Hippel und Fleige 2020). Programm- und bildungstheoretisch gilt entsprechend: „Ein Programm ist der zeitgeschichtlich materialisierte Ausdruck gesellschaftlicher Auslegung von Erwachsenenbildung durch einen bestimmten Träger, realisiert über eine Vielzahl an Angeboten. Es ist beeinflusst durch bildungspolitische und ökonomische Rahmungen (…) und potenzielle Adressat/innen. Es wird ausgelegt und gefiltert durch professionell Handelnde“ (Gieseke 2015, S. 163). In dem relativ offenen und ungeregelten Bereich der Erwachsenen- und Weiterbildung lassen sich über die Programme Themen- und Aufgabenstrukturen (nach Programmbereichen und darüber hinaus), Zielgruppenstrukturen, aber auch Organisationsprofile der Anbieterinnen fassen und rekonstruieren, Entwicklungen im Zeitverlauf und Ausdifferenzierungen (auch im Hinblick auf die derzeit sich entwickelnden beigeordneten Bildungsangebote dritter Institutionen).

*Programmforschung* umfasst die Erforschung der Programme und Angebote, wie auch des Programmplanungshandelns. Dazu ist in den vergangenen drei Jahrzehnten im deutschsprachigen Raum eine Reihe von empirischen und theorieentwickelnden Studien vorgelegt worden, teilweise hinterlegt mit (größeren) Drittmittelprojekten und Konsortien – gefolgt von Übersichtspublikationen zu Fragestellungen, Zugängen, Verfahren und Ergebnissen der Programmforschung, wie: einem Lehrbuch von Fleige et al. (2019); Zeitschriftenausgaben (Hess. VHS-Verband 2/2019; BV Päd. 4/2020; DGWF 02/2021), Handbuchtexten (u. a. Schrader und Ioannidou 2011) und Handreichungen. Auch in anderen Ländern existieren Programmforschungstraditionen und es beginnt ein international-vergleichender Diskurs (z. B. Daffron und Caffarella 2021; Käpplinger et al. 2017). Diese und weitere Publikationen sowie auch die o. g. Originalstudien sind verzeichnet in der zentralen Bibliografie der Homepage der Expertinnengruppe Programmforschung (https://die-bonn.de/li/250).

Bislang eher in den deutschsprachigen Ländern entwickelt ist die Tradition der Programmarchive – ausgehend vom Volkshochschulprogrammarchiv am DIE, dem Programmarchiv des Österreichischen Volkshochschularchivs und dem regional-trägerübergreifenden Weiterbildungsprogrammarchiv Berlin/Brandenburg an der Humboldt-Universität zu Berlin. Programme und Angebotsankündigungen als Datentypus unterscheiden sich dabei von Anbieterstatistikdaten, wie sie für das Bildungsmonitoring herangezogen werden; dies u. a. dadurch, dass sie geplante und nicht bereits realisierte Angebote abbilden und mit studienbezogenen Untersuchungskategorien auswerten (Fleige und Reichart 2014). Anbieterdatenstatistiken und Programmdaten lassen sich aber auch aufeinander beziehen, etwa indem Statistikdaten die Sample- oder Kategorienbildung für Programmforschung anregen (so u. a. aktuell bei Fleige et al. 2022).^1^

Die Heftnummer will Stand und Entwicklungen der Programmforschung einordnen, Systematisierungen fortschreiben und anhand aktueller Forschungsbeiträge verdeutlichen. Es soll aufgezeigt werden, welche Fragestellungen in einer Zeit (der Transformation) an die Programmforschung herangetragen werden, welche theoretischen Zugänge, Verfahren und Methoden herangezogen und ggf. verschränkt werden, und was die Programmforschung zur Ausdifferenzierung des Wissens über Erwachsenen- und Weiterbildung und über das professionelle Handeln in diesem Bildungsbereich beiträgt.

Nun zeigt die Forschungsgenese, dass sich innerhalb der Programmforschung vor allem die methodischen Zugänge zu den im engeren Sinne als *Programmanalysen* (bzw.* Angebotsanalysen*) zu bezeichnenden Untersuchungen ausdifferenziert haben (auch Gieseke und Robak in diesem Heft). Programmanalysen nehmen Bezug auf das Programm als Spezifikum der Erwachsenenbildung. Um über Programmanalysen grundlegende Aussagen über die Aufgaben und Organisationen der Erwachsenen- und Weiterbildung zu treffen, werden komplexe Designs und Instrumente angewendet. Zusätzlich werden inzwischen elektronische Datenbanken und Repositorien von insbesondere Programmarchiven in die Programmforschung eingebracht, mit digitalen Suchfunktionen und Möglichkeiten zur Korpusbildung – ebenso wie digitale Analysetools (Freide et al. 2020; Käpplinger und Dietz 2021; Robak et al. 2023 i. V.). Vor diesem Hintergrund bemüht sich die Heftnummer in* methodentheoretischer *Hinsicht besonders um die *Einordnung von Programmanalysen* bzw. *von Forschung mit Programmen und Angeboten*. Wir schlagen nachfolgend eine Systematisierung vor, in welche sich bereits a. a. O. vorgestellte Untersuchungen und die in diesem Heft vorgestellten aktuellen Studien einordnen lassen. Zentral sind hier Differenzkategorien für unterschiedliche Arten und Zugänge von Programmanalysen wie in der Methodendiskussion eingeführt (vgl. Käpplinger 2011; Nolda 2018; Robak 2012; Fleige et al. 2019) und hier fortgeschrieben. Unsere Kategorien können dabei unabhängig voneinander und in Kombination miteinander auf Untersuchungen angewendet werden (Tab. 1).

**Table 1:** 

Differenzkategorie …	… auf einem Kontinuum
A) Kodier‑/Auswertungsprozess	A1) Kodierung von Programmen/Angeboten anhand eines langen (in einem abduktiven Prozess entwickelten) Kodebuchs *(die Anzahl der für die Analyse ausgewählten Angebote ist dabei nicht abhängig von der Länge des Kodebuchs);* A2) Kodierung von Programmen/Angeboten anhand eines kurzen Kodebuchs und ggf. mit Unterstützung von digitalen Wortsuchfunktionen im Rahmen von Datenbanken; A3) Kodierung von Programmen/Angeboten mit digitalen Tools ohne Kodebuch (mit der Hilfe von Suchfunktionen innerhalb von Angebotsdatenbanken in Repositorien, siehe Kategorie C, oder als Big Data Analysis, Topic Modelling etc.)
B) Angebots‑/Programmbezug	B1) Angebote werden für sich stehend untersucht B2) Angebote werden auch als Teil von einem Programm einer Institution untersucht
C) Herkunft und Art der Präsentation von Forschungsdaten	C1) aus Programmarchiv oder von Weiterbildungsanbietern (Weiterbildungsinstitutionen, beigeordnete Anbieter, Verbände) (jeweils print oder digital) (digital über: Pdf-Dateien von Programmheften auf Homepages der Institutionen, Datenbanken zur Anmeldung)^b^ C2) digitale Repositorien von in Print bestehenden Programmarchiven oder digitale Repositorien ALS Programmarchiv C3) aus Weiterbildungsdatenbanken dritter Anbieter (Angebotsdatenbanken, nicht zu Sammlungszwecken, sondern zu Veröffentlichungs- und Anmeldungszwecken)
D) Funktion der Angebots‑/Programmanalyse im Forschungsprozess	D1) Vorbereitende Exploration D2) Sample‑/Korpusbildung D3) Untersuchung zur Ausdifferenzierung
*Quer dazu liegend:* Forschungsverfahren und Methoden zur Auswertung (nach Kodierung)	E1) Quantitativ/statistisch (vermittels unterschiedlicher Erfassungs- und Auswertungstools und für unterschiedlich große Datensätze)^c^ E2) Qualitativ-hermeneutisch E3) Kombination^d^

^a^Wir danken B. Käpplinger und S. Robak für Austausch und Anregungen in Bezug auf diese Systematik.

^b^Programmforschung kann mit gedruckten Programmen wie mit in unterschiedlicher Weise digital vorliegenden Programm- und Angebotsankündigungen arbeiten. Bei der aktuellen Ausdifferenzierung der Formen der Angebotsankündigung und Programmveröffentlichung gilt gleichwohl (weiterhin), dass das gedruckte Programm stärker als Newsletter und Einrichtungsdatenbanken die Funktionen der Bedarfsweckung und Zielgruppenerreichung erfüllt (vgl. Käpplinger 2021), so dass nicht per se von einem ‚Verschwinden des Programmheftes‘ auszugehen ist.

^c^Bislang werden hier vor allem uni- und bivariate Analysen eingesetzt und könnten multivariate Auswertungsverfahren stärker berücksichtig werden.

^d^Nach der qualitativ-quantifizierenden und deskriptiv-statistischen Analyse werden weitere qualitativ-interpretative Auswertungsschritte vorgenommen: Auffälligkeiten in den Programmstrukturen werden entlang theoriegeleiteter Fragestellungen und nochmals induktiv-erschließend anhand desselben Datenmaterials vertiefend-interpretativ bearbeitet (aktuell weitergehend in Robak et al. 2023 i. V.).

Aus den Kombinationen dieser Differenzkategorien ergeben sich *verschiedene Arten/Zugänge von Untersuchungen zu Angeboten und Programmen*. Als *Programmanalysen* im engeren Sinne würden wir allerdings nach jetzigem Stand der Diskussion und Begriffsbildung *programmbezogene Untersuchungen mit Kodebüchern* (als zentralem Kriterium) bezeichnen. Gleichwohl finden sich in dieser Heftnummer auch Beiträge, die andere Verfahren anwenden. Insbesondere deren Bezug zu Programmarchiven rückt sie dabei in eine besondere Nähe zu Programmanalysen, machen sie zu einem weiteren Zugang zur Programmforschung.

## Zu den Beiträgen im Schwerpunkt

Der Fokusbeitrag von *Wiltrud Gieseke* und *Steffi Robak* „Bildungsforschung als Zugang um gesellschaftliche Wirklichkeit zu erschließen“ leitet die Programmforschung forschungshistorisch umfassend her und verbindet diese Betrachtungen mit methodentheoretischen Reflexionen sowie Betrachtungen zu Kodebüchern als methodischem und gegenstandstheoretischem Grundbezugspunkt von Programmanalysen. Dabei wird auch die Bedeutung unterschiedlicher Studien als Beitrag zur Ausdifferenzierung des Wissens über Erwachsenen- und Weiterbildung in der jeweiligen historischen Situation und diachron herausgearbeitet. Die Autorinnen verdeutlichen damit auch erkenntnistheoretische Anknüpfungspunkte für andere Disziplinen.

Es folgen fünf Originalbeiträge zu aktuellen Untersuchungen – die entweder Angebote und Programme mit Programmanalysen, Angebotsanalysen oder mit digitalen Tools wie Text Mining-Verfahren analysieren (Käpplinger; Nylander und Holmer; Scheidig) oder das Programmplanungshandeln auf der Grundlage von Interviews und Gruppendiskussionen untersuchen (Haberzeth und Dernbach-Stolz; Kulmus) und die Befunde methodisch, theoretisch und thematisch diskutieren.

Drei der fünf Beiträge eröffnen Zugänge zur Forschung über Programme und Angebote, zwei legen dabei Programmanalysen im o. g. engeren Sinne vor, und wiederum zwei nutzen Programmarchive bzw. Repositorien. Der Fokus liegt auf dem Organisationstypus Volkshochschule und seinen Programmstrukturen unter einer spezifischen, jeweils neuen Fragestellung. Im Spiegel zeithistorischer Anforderungen an die Programmplanung machen drei der fünf Beiträge Aussagen zur Gestaltung von Programmen und Angeboten unter den Bedingungen der Coronapandemie. Diese sehr zeitaktuellen Beiträge stehen wiederum im Kontrast zu zwei anderen Beiträgen, die historisch, längsschnittlich und vergleichend auf Programme blicken.

Der Artikel von* Bernd Käpplinger* „Arbeitspläne und Leitideen in der Weimarer Republik – Divergente Richtungen der Volkshochschulen in Bremen und in Gießen“ ist der historischen Programmforschung zuzurechnen. Der Beitrag gibt einen Überblick über historische Arbeitsplan- und Programmanalysen insbesondere mit dem Fokus auf Volkshochschulen. Er kontrastiert empirisch mit einer qualitativen Programmanalyse die Arbeitspläne der Volkshochschulen Bremen und Gießen mit einem Schwerpunkt auf den Vorworten bzw. Leitbildern während der Weimarer Republik und arbeitet detailliert sehr interessante Differenzen heraus. Darüber hinaus diskutiert er inwiefern Arbeitspläne bzw. Programme (mehr als) Angebotskommunikationen sind und zeigt die Vielfalt der historischen Leitideen der Volkshochschulen auf.

*Erik Nylander* und *Daniel Holmer* führen in „Seismographs to Society? Tracing Topics from Swedish folk high schools through Large-scale Text Analysis 1954–2007“ das neuetablierte Programmarchiv zu einem schwedischen Typus von Volkshochschulen (*Folkhögskola*) ein, welches als digitales Repositorium entstanden ist und verdeutlichen dessen forschungstheoretische und historiographische Bedeutung. Ihre einfließende Parallelbetrachtung zur Volkshochschularbeit in Deutschland öffnet für einen vergleichenden Blick. Die Autoren verknüpfen den Aufbau des Repositoriums mit einer Perspektive für Programmforschung unter Anwendung großangelegter Textanalysen mit digitalen Tools und präsentieren Topic Modelling-Ergebnisse. Die längsschnittliche Analyse setzte es sich zum Ziel, Veränderungen von Themen und Kursbeschreibungen aufzuspüren und im Kontext von Veränderungen im schwedischen System der Erwachsenen- und Weiterbildung (demokratiebildungsbezogenen) zu interpretieren.

*Falk Scheidig* untersucht „De(n) ‚Corona-Schock‘ als Digitalisierungsschub? Eine vergleichende Programmanalyse zu Angeboten politischer Erwachsenenbildung an Volkshochschulen unmittelbar vor und während der Pandemie“. Den Autor interessiert, ob sich das Verhältnis von Online- und Präsenzveranstaltungen durch die Pandemie verändert hat, dies speziell in der Politischen Bildung. Um hier eine größere Anzahl von Programmen bzw. Ankündigungen untersuchen zu können, nutzt er das digitale bzw. digitalisierte VHS-Programmarchiv am DIE und diskutiert dessen Bedeutung für die Forschung (sowie die Wechselwirkungen mit den Veröffentlichungswegen (print, digital) für Programme). Der Autor wertet Programme von dreißig VHS quantitativ aus (und beschaffte für diese Stichprobe aktuelle Anschlussprogramme selbst) und relationiert Unterrichtsstunden und Formate. Die Ergebnisse geben Hinweise für weitergehende Forschung, da sich noch keine klaren Entwicklungsrichtungen ausmachen lassen.

*Erik Haberzeth* und *Stefanie Dernbach-Stolz* fragen in „Programmplanung in der Weiterbildung unter dem Einfluss der Corona-Pandemie: Resultate einer empirischen Studie zu neuen Anforderungen“ danach, wie sich die Programmplanung durch die Corona-Pandemie verändert hat. Hierzu haben sie 10 Gruppendiskussionen mit Planenden geführt und vor dem Hintergrund des Modells der Wissensinseln (Gieseke) ausgewertet. Als Wissensinseln fokussiert haben sie Bedürfnis- und Zielgruppenanalyse sowie die Angebotsentwicklung und arbeiten heraus, wie wichtig diese für die in ihrer Bedeutung gestiegene Profilbildung sind. Besonders interessant in den Ergebnissen ist auch die Verdeutlichung der gestiegenen und ausdifferenzierten Anforderungen an das professionelle Handeln der Planenden, insbesondere in den Bereichen des Entscheidens und Begründens des eigenen Handelns, was unter anderem den Kern professionellen Handelns ausmacht.

*Claudia Kulmus* betrachtet in „Seniorenbildung in der Pandemie: Programmplanung zwischen Erstarrung und Innovation“ in einer Interviewstudie Planungsstrategien in der Pandemie in Einrichtungen mit Bildungsangeboten für Ältere. Hierbei unterscheidet sie vier traditionelle Bildungsorte für Ältere (VHS, universitäre Einrichtung, kirchliche Einrichtung, kommunale Seniorenbegegnungsstätte). Besonders interessant ist an den Ergebnissen die Herausarbeitung von vier Planungsstrategien entlang von zwei Dimensionen (Wissensvermittlung – Begegnung, Lähmung – Innovation). Bei den Planungsstrategien werden so sichtbar: eine kontinuierliche digitale Innovation, eine strukturelle Innovation, ein abwartendes Situationsbeobachten, ein Wunsch nach Rückkehr zum Alten. Theoretisch schließt die Autorin dabei hier an Modellbildungen zu Planungsstrategien innerhalb der Forschung zum Programmplanungshandeln (Gieseke, Pohlmann, Lorenz) an.

Die hier versammelten Artikel ergeben z*usammen mit weiteren aktuellen Beiträgen der Programmforschung* (siehe die o. g. Online-Bibliografie unter https://die-bonn.de/li/260) einen aktuellen Einblick in diese Forschungsrichtung.

## Zu den Beiträgen im Forum

Der Artikel von *Sarah Widany, Elisabeth Reichart, Nicolas Echarti und Kerstin Hoenig* fokussiert auf der Grundlage einer Auswertung aktueller WB-Monitor-Daten (Sonderbefragung) und der VHS-Statistik die Entwicklung von Programmstrukturen während des ersten Corona-Lockdowns und diskutiert sie im Hinblick auf ihre Bedeutung für den Bereich der Erwachsenen- und Weiterbildung. Die Befunde eines Angebotsrückgangs einerseits und einer Zunahme an Einzelveranstaltungen andererseits bedürfen dabei, so die Autorinnen und der Autor, weitergehender Untersuchungen – auch unter Hinzunahme von Programmanalysen.

WB-Monitor-Daten von 2017 nutzend, fragen *Martin Reuter, Caroline Dietz und Laureen Fröhlich* nach „Pädagogik, Legitimation und Ressourcen im Kontext von Qualitätsmanagementsystemen – Herausforderungen in organisationalen Feldern der Weiterbildung aus neo-institutionalistischer Perspektive“. Sie unterscheiden diese für vier Typen von Weiterbildungsanbietern und analysieren inhaltsanalytisch Unterschiede und Gemeinsamkeiten in den Auswirkungen und Handhabungen von Qualitätsmanagementsystemen entlang der Kategorien „Legitimation“, „Pädagogik“ und „Ressourcen“, welche sie neoinstitutionalistisch ableiten.

Der Beitrag von *Annika Felix, Sarah Berndt, Jasmin Dabitz und Paul Schubert* bezieht sich wiederum auf die Erwachsenen- und Weiterbildung unter pandemischen Bedingungen, hier im Spiegel der Teilnehmendenforschung. Die Autorinnen und der Autor fragen nach „Wissenschaftliche(r) Weiterbildung Älterer in Zeiten der COVID-19-Pandemie – Sichtweisen von Teilnehmenden an ‚Studieren ab 50‘ der Universität Magdeburg“. Dazu wurden Teilnehmende des Jahres 2021 befragt. Mittels Regressionsanalysen wird beschrieben, wie sich das Teilnahmeverhalten in der Pandemie änderte und welche Faktoren dabei eine Rolle spielten (auch Zusammenspiel bisheriger Teilnahmeerfahrungen und neuer Faktor Kontaktangst). Die Ergebnisse werden im Hinblick auf zukünftige Programmgestaltungen diskutiert.

Eine Rezension zu „Erich Schäfer, Antje Ebersbach: Die digitale Transformation in der Weiterbildung. Befunde, Konzepte und Perspektiven“ von *Sophie Lacher* rundet die Heftnummer ab.

Wir bedanken uns bei allen Autorinnen und Autoren sowie Gutachterinnen und Gutachtern für ihre Beiträge und wünschen allen Lesenden interessante Einblicke.

## Footnotes

1 Dabei gilt: Programme lassen gegenüber Statistiken die Bildungsauslegungen und -anforderungen in einer Zeit rekonstruieren und wollen nicht evaluieren, welche Kurse erfolgreich angeboten werden. Es wäre gleichwohl wünschenswert, für das Monitoring von Programmstrukturen auch Programmdaten hinzuzuziehen, auch i. S. der bereits stattfindenden Ausdifferenzierung einbezogener Datentypen.
